# Convergent Pathways in Idiopathic Autism Revealed by Time Course Transcriptomic Analysis of Patient-Derived Neurons

**DOI:** 10.1038/s41598-018-26495-1

**Published:** 2018-05-30

**Authors:** Brooke A. DeRosa, Jimmy El Hokayem, Elena Artimovich, Catherine Garcia-Serje, Andre W. Phillips, Derek Van Booven, Jonathan E. Nestor, Lily Wang, Michael L. Cuccaro, Jeffery M. Vance, Margaret A. Pericak-Vance, Holly N. Cukier, Michael W. Nestor, Derek M. Dykxhoorn

**Affiliations:** 1 John P. Hussman Institute for Human Genomics, University of Miami Miller School of Medicine, Miami, Florida 33136 USA; 2 Department of Medical & Molecular Genetics, Oregon Health & Science University, Portland, Oregon, 97239 USA; 3 The Hussman Institute for Autism, Baltimore, Maryland 21229 USA; 4 John T. Macdonald Foundation Department of Human Genetics, University of Miami Miller School of Medicine, Miami, Florida 33136 USA; 5 Department of Neurology, University of Miami Miller School of Medicine, Miami, Florida 33136 USA; 6 Department of Public Health Sciences, University of Miami Miller School of Medicine, Miami, FL 33136 USA

**Keywords:** Induced pluripotent stem cells, Autism spectrum disorders

## Abstract

Potentially pathogenic alterations have been identified in individuals with autism spectrum disorders (ASDs) within a variety of key neurodevelopment genes. While this hints at a common ASD molecular etiology, gaps persist in our understanding of the neurodevelopmental mechanisms impacted by genetic variants enriched in ASD patients. Induced pluripotent stem cells (iPSCs) can model neurodevelopment *in vitro*, permitting the characterization of pathogenic mechanisms that manifest during corticogenesis. Taking this approach, we examined the transcriptional differences between iPSC-derived cortical neurons from patients with idiopathic ASD and unaffected controls over a 135-day course of neuronal differentiation. Our data show ASD-specific misregulation of genes involved in neuronal differentiation, axon guidance, cell migration, DNA and RNA metabolism, and neural region patterning. Furthermore, functional analysis revealed defects in neuronal migration and electrophysiological activity, providing compelling support for the transcriptome analysis data. This study reveals important and functionally validated insights into common processes altered in early neuronal development and corticogenesis and may contribute to ASD pathogenesis.

## Introduction

The genetics underlying ASD are complex and highly heterogeneous, with over 400 genetic loci of various levels of confidence implicated to date^1–3^. In addition to genetic heterogeneity, there is considerable phenotypic heterogeneity with a variety of deficits in adaptive function, cognition, and language ranging widely between affected individuals. Hence, ASD is comprised of a “spectrum” of disorders which present with a range of severity.

Research efforts have focused on understanding the biological basis of ASD and significant progress having been made in the identification of genetic risk factors associated with this disorder. Despite these advances, gaps remain in our understanding of the molecular and cellular mechanisms that underlie the observed ASD clinical phenotypes. Independent results from a number of recent studies indicate that a large proportion of ASD-associated genes are functionally interconnected through pathways that control a broad range of processes during brain development, including transcriptional regulation, neuronal differentiation and cell fate specification, neural connectivity and function, cell adhesion and migration, and maintaining the balance between excitation and inhibition^4–6^.

Induced pluripotent stem cells (iPSCs) have emerged as a powerful tool for modeling a variety of human diseases, including genetically complex psychiatric disorders^7–11^. iPSC lines can be generated directly from clinically defined patients, allowing the complete genetic profile of the individual to be replicated in the iPSC line, irrespective of whether the pathogenic alteration is identified in advance, with genetic backgrounds that are known to result in the development of ASD^12^. The *in vitro* differentiation of human iPSCs into regionally-specific, well-defined, subtypes of neurons mimics the same neurodevelopmental processes that occur during *in vivo* neurogenesis, when regional patterning is taking place in the developing cerebral cortex^13^. This makes iPSC-derived neurons well-suited for modeling human neurodevelopmental conditions such as ASD as they offer an experimental platform for studying the cellular and molecular mechanisms that give rise to the disorder, and that underlie its progression at the onset of pathogenesis during prenatal and early postnatal brain development. This can be accomplished by examining the biology of ASD in iPSC-derived neurons at various stages in their cellular development and functional maturation.

ASD is a genetically complex disorder where no one gene has been found to be responsible for more than ~1% of ASD patients. RNA-Seq provides a comprehensive and unbiased approach to identify differentially expressed transcripts between affected and unaffected individuals, including those related to low-copy transcripts, novel transcripts, non-coding RNAs, and splice variants that could underlie the biology of ASD^14^. Evidence indicates that dysregulation of gene expression patterns, copy number variations, splicing and long non-coding RNA could influence ASD susceptibility. However, most of these studies used either immortalized cell lines or postmortem brain tissue which each have limitations in how efficiently they model neurodevelopmental disorders such as ASD^5,15–22^. Recent studies have indeed attempted to investigate the biology of ASD by analyzing the transcriptome of iPSC-derived neurons, including studies of idiopathic autism^23,24^ and iPSC models where key candidate genes were disrupted by genetic engineering approaches^8,25–27^.

In this study, we aim to identify common cellular and molecular signatures that distinguish iPSC-derived neurons from our cohort of idiopathic ASD individuals from those of unaffected controls, and evaluate how those signatures change over the course of neuronal development. We approached this by identifying expression differences in neurons derived either from individuals with ASD or controls, both on a single gene and higher-order network level. Transcriptional analyses of the ASD and control neurons showed ASD-specific molecular phenotypes affecting networks involved in neuronal differentiation, the cytoskeletal matrix structure formation, patterning, DNA and RNA metabolism. We then applied functional analyses to identify disease-specific, cellular phenotypes. Functional analysis of the ASD-derived neurons demonstrated deficits in neuronal activity and cell migration, hence providing compellingly support for the transcriptome analysis data. Taken together, both transcriptional and functional misregulation is occurring during neuronal development in our patient-derived models of idiopathic ASD.

## Results

### Derivation of ASD patient-specific iPSC lines and cortical neuron differentiation

Six male non-Hispanic white (NHW) individuals with idiopathic ASD were selected for iPSC reprogramming (Table 1). Fresh blood was collected from these patients as well as from two control individuals, peripheral blood mononuclear cells (PBMCs) isolated, and stem cell reprogramming performed. These lines were analyzed for pluripotency (Suppl. Figure 1) and karyotypic stability (Suppl. Figure 2). In addition, genetic variants that had been previously identified in the patients through copy number variation (CNV) analysis^28^ or whole exome sequencing^29,30^ were confirmed in the patient iPSC lines by Sanger sequencing (Suppl. Figure 3). Two of the controls were derived from PBMCs isolated from cognitively normal NHW male individuals (Control114 and Cont574) and reprogrammed as above. The additional controls were derived from (1) cardiac fibroblasts from a cognitively normal NHW male (Cont1021) and (2) foreskin fibroblasts from NHW male individuals (Cont101 and Cont102). All iPSC, both ASD and control, were differentiated according to the following protocol. For differentiating neurons with relevance to ASD, several important criteria were considered, including: (1) accurate recapitulation of the neurodevelopmental process, (2) brain region specificity, and 3) neuronal cell type. Potential neurodevelopmental mechanisms altered in ASD include neuronal migration in the developing cerebral cortex and abnormal cortical connectivity^31–35^. Thus, for investigation of altered neurodevelopmental mechanisms in ASD, we optimized a differentiation protocol (Suppl. Table 1) that mimics cortical neurogenesis (Suppl. Figure 4). A schematic of this protocol is outlined in Supplemental Fig. 4A and described in the Methods section. To optimize this protocol, we examined the expression of key neural progenitor and neuronal markers throughout the differentiation procedure. Cells within neural rosette structures were shown to express Nestin (Suppl. Figure 4B) and SOX2 (Suppl. Figure 4B), which are standard markers for neural stem cells. Neural rosettes were selected and plated on PLO/L plates. Doublecortin (*DCX*; a marker for immature migrating neurons) expression was analyzed between days 12 and 90 and found to coincided with increased expression of calcium/calmodulin-dependent protein kinase II α (*CAMK2A*; a marker for excitatory cortical neurons). As expected, we found a gradual rise in *DCX* expression that peaked at day 60 in culture and showed signs of falling by day 90 (Supplemental Fig. 4C and D). Notably, our observation that iPSC-derived neurons show decreased expression of *DCX* at day 90 (23 weeks post-neural induction) is in alignment with *in vivo* data available on BrainSpan (BrainSpan Atlas of the Developing Human Brain; http://brainspan.org/) which indicates that *DCX* expression tapers off in the developing human cortex after 24 weeks post-conception. As expected, *CAMK2A* expression levels showed a gradual increase over a period of 12 to 90 days in culture (Supplemental Fig. 4C). At day 25, both control and ASD neurons showed robust expression of TBR1, a marker of glutamatergic projection neurons (Supplemental Fig. 4D). By day 90, dense neural networks staining positively for MAP2 and SYNAPSIN 1 were seen in the culture. These results validate this differentiation approach and show that it enriches for excitatory glutamatergic neurons.

**Table 1:** Clinical description of ASD patients used for iPSC generation.

Case #	Gender	Diagnosis	Developmental Issues	Neurologic and Neuropsychiatry Features	Medical Conditions	Head Circumference	Cognitive Functioning	Adaptive Functioning	Candidate Gene (s)
709	M	ASD	Developmental Delay	None Reported/Found in MR	Asthma	Normocephalic	—	Low	RBFOX1
110	M	ASD	Mild Hypotonia	None Reported/Found in MR	Pituitary Dwarfism/Growth Hormone Deficiency	>95%	—	Low Average	VPS13B, EFCAB5, TRIM55
691	M	ASD	Language Delay	None Reported/Found in MR	—	Normocephalic	Very Low	Moderately Low	COL6A3, SLIT3, C2orf85, AB13BP, UIMC1
134	M	ASD	Phonological Disorder	ADHD/Neuronal Migration Disorder/Possible Seizures/Sleep Problems	Eczema	Normocephalic	Average	Average	CPZ, PRICKLE1, TOPOR5
725	M	ASD	None Reported/Found in MR	ADHD-Inattentive Type	Hydrocele/Hernia	Normocephalic	—	Moderately Low	SOS2, IRMVI55, ZMYND17, BTN2A2, MDC1, FBXO40, KIAA1949
732	M	ASD	Language Regression at Age 28m	None Reported/Found in MR	Acid Reflux/Allergies	Normocephalic	—	—	CLCN2, F13A1, JARID2, STXBP5, C12orf73, C20orf118, FGD6

The dashed lines represent characteristics that were either not assessed or impossible to ascertain from the available clinical file.

### Time course transcriptomic analysis

**Figure 1:**
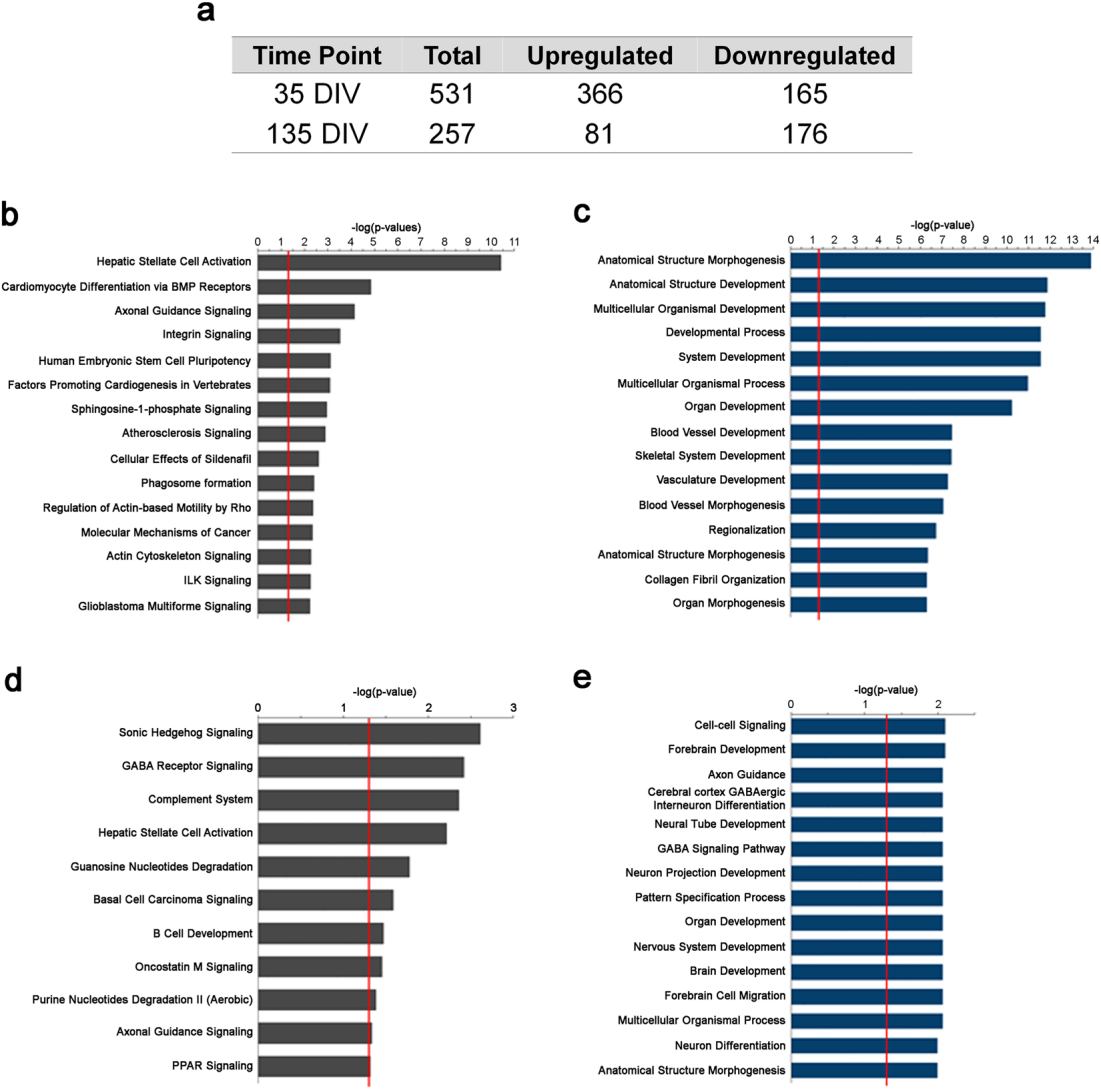
Time course transcriptome analysis of genes differentially expressed between ASD and control iPSC-derived neurons. (**a**) Table reflecting the number of total, upregulated, and downregulated significantly differentially expressed genes (FDR <0.05) between ASD and control neurons at each time point. (**b**) Top 15 IPA pathways enriched with day 35 DEGs. (**c**) Top 15 GO biological processes enriched with day 35 DEGs. (**d**) Top IPA pathways enriched with day 135 DEGs. (**e**) Top 15 GO biological processes enriched with day 135 DEGs. The red line in the bar plots indicates the cut-off for significance (adjusted P = 0.05).

To gain insight into pathways disrupted by ASD during cortical development, and to better understand how these disturbances to cellular function progress as neurons mature, we performed RNA-Seq on ASD and control neurons at two time points after neural induction (35 and 135 days *in vitro*; DIV), then analyzed the data using multiple approaches (Suppl Figure 5). We chose these two time points to capture differences in the transcriptome at early stages of development (late neuronal progenitor/early neuron stage (Day 35)) and later stage where there are more mature neurons (Day 135). Hierarchical clustering of the samples at each time point showed that the ASD samples segregated into a distinct group from the control samples (Suppl. Figure 6A,B). Individual genes with significant differential expression between ASD and control neurons were identified at each time point using EdgeR, then further analyzed via Ingenuity® Pathway Analysis (IPA) and the Cytoscape^36^ Plugin BiNGO^37^ to discern the potential functional pathways and biological processes affected. Differential expression analysis of ASD and control transcriptomes at the two points in our time course identified 531 differentially expressed genes (DEGs; FDR <0.05) at 35 DIV and 257 DEGs at 135 DIV (Fig. 1a, Suppl. Data 1 and 2).

IPA analysis of the differentially expressed genes at 35 DIV indicated a dysregulation in functional pathways mainly pertaining to cytoskeletal protein signaling, axon guidance, motility and differentiation (Fig. 1b). Moreover, gene ontology (GO) enrichment analysis of the DEGs at 35 DIV indicated a dysregulation in biological processes mainly pertaining to anatomical structure development and morphogenesis, regionalization and collagen fibril organization (Fig. 1c). IPA analysis of DEGs in ASD neurons at 135 DIV indicate altered inhibitory neuron development and function (i.e. dysregulation of pathways involving Sonic Hedgehog and GABAR signaling, respectively). 135 DIV DEGs were also enriched for pathways mediating nucleotide metabolism, cytoskeletal protein signaling, and axon guidance (Fig. 1d). GO analysis of DEGs at 135 DIV show enrichment in biological processes related to nervous system anatomical development and morphogenesis, brain patterning, neuronal differentiation, neuronal projection development, and axon guidance (Fig. 1e).

**Figure 2:**
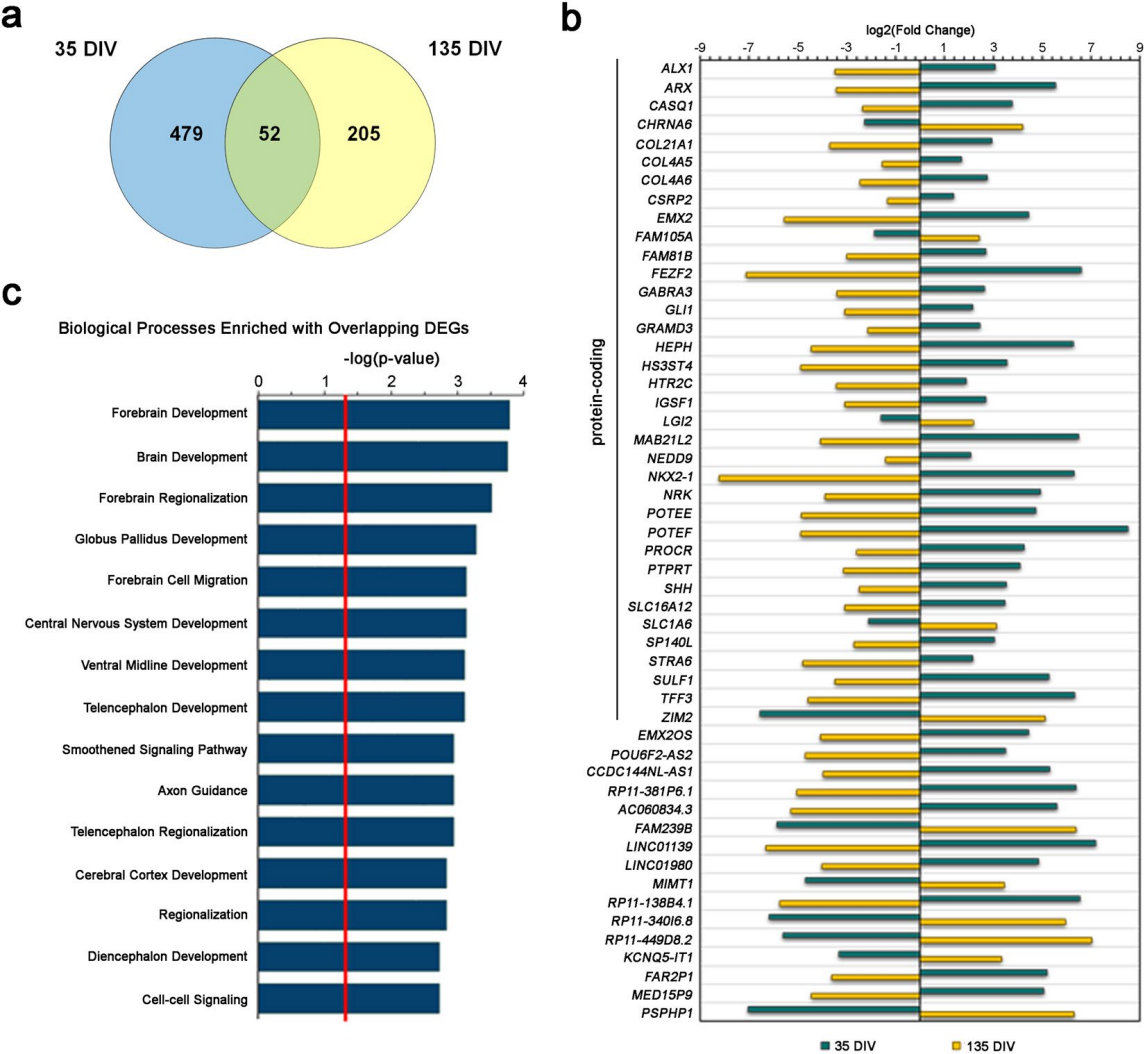
Overlap in genes differentially expressed between ASD and control neurons at 35 and 135 DIV. (**a**) Venn diagram reflecting the number of unique and overlapping significantly (FDR <0.05) differentially expressed genes at each of the time points. (**b**) Bar plot showing Log2 fold change of overlapping DEGs across 35 and 135 DIV time points. (**c**) Top 15 GO biological processes enriched in DEGs shared between day 35 and 135 time points. The red line in the GO biological processes bar plot indicates the cut-off for significance (adjusted P = 0.05).

There were 52 genes with FDR <0.05 at both the 35 and 135 DIV time points (Fig. 2a,b). This gene expression data from DEG overlap between time points were analyzed via IPA and BiNGO to discern the potential functional pathways and biological processes affected (Suppl. Figure 5). Interestingly, all overlapping DEGs showed a reversal of expression patterns when comparing the 35 DIV time point to the 135 DIV time point (Fig. 2b). Additionally, GO analysis demonstrated an enrichment in biological processes pertaining to brain development, regionalization and axon guidance (Fig. 2c).

In addition, GO analysis of DEGs was performed against two different neuronal background sets: 1) genes expressed across all of our iPSC-derived neuronal lines and (2) genes expressed in human fetal brain over the course of prenatal development (obtained from BrainSpan^38^). The analyses of DEGs against these gene sets show that both neuronal background sets yield nearly identical results (Suppl. Figure 7) to each other and to those from our original GO analysis of DEGs using the whole human genome background set (Figs 1c,d and 2c). These include the overrepresentation of genes involved in generalized developmental pathways (for example, anatomical structure morphogenesis and development and system development) at 35 DIV and forebrain development, neuron projection development, and axonogenesis at 135 DIV.

**Figure 3:**
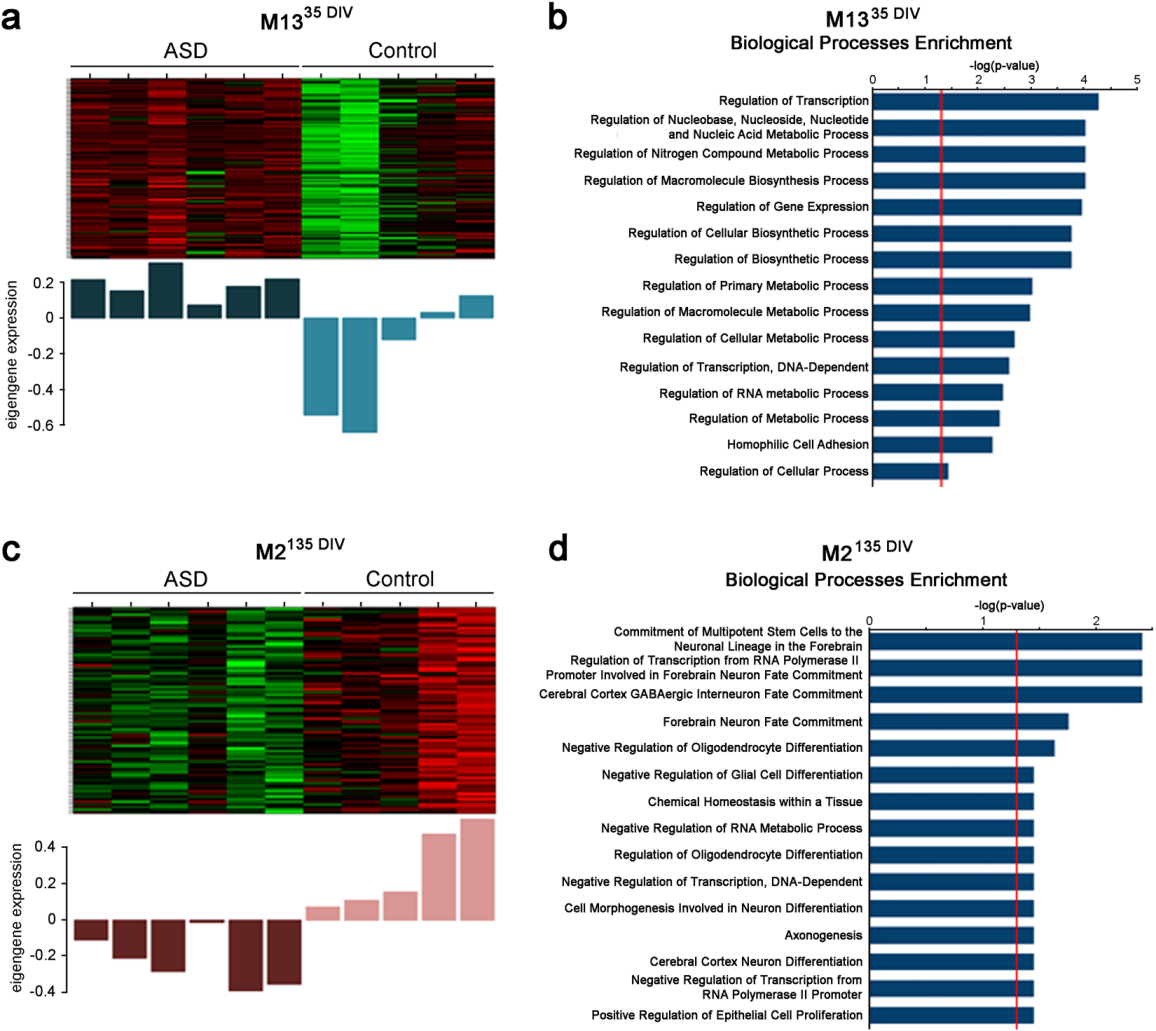
Co-expression network analysis identifies modules associated with ASD at early and later stages of neuronal development *in vitro*. Heatmaps and GO analysis of representative ASD correlated WGCNA modules from each time point. (**a,c**) Heatmaps showing ASD and control neuron expression levels for genes represented in the day 35 module, M13d35 (**a**) and day 135 module, M2d135 (**c**). The bar plots displayed underneath the heat maps show module eigengene (1st principal component) across subjects. (**b,d**) Top 15 GO biological processes enriched with genes belonging to M13d35 (**b**) and M2d135 (**d**). The red line in the GO biological processes bar plot indicates the cut-off for significance (adjusted P = 0.05).

Next, a systems-level approach to identifying patterns of gene co-expression from RNA-Seq data was utilized to identify potential patterns of biologically relevant cellular mechanisms disturbed by disease^39^. Therefore, weighted gene co-expression network analysis (WGCNA)^40–42^ was performed (Suppl. Data 3). WGCNA analysis yielded a total of 49 modules with significant correlation to the ASD phenotype (p < 0.05). Specifically, 23 modules correlated with ASD neurons at 35 DIV and 26 modules for 135 DIV. Genes belonging to each module correlated with ASD were fed into the IPA and GO pipelines to identify enrichment in specific functional pathways and biological processes. Surprisingly, all 49 of the ASD correlated modules presented enrichment in IPA functional pathways (Suppl. Data 4 and 5). Regarding modules correlated with ASD at day 35, M16^d35^ was enriched with embryonic stem cells differentiation and transcriptional regulatory network pathways. The M4^d35^ module was enriched in actin cytoskeleton signaling, motility and axon guidance pathways. The M3^d35^ module was enriched with glutamate receptor signaling, calcium signaling and other multiple biosynthesis, degradation and metabolic pathways. As for GO analysis, only nine 35 DIV WGCNA modules presented enrichment in GO biological processes (Suppl. Data 6). For example, M13^d35^ showed enrichment in processes pertaining to regulation of gene expression and cellular metabolic processes including DNA and RNA metabolism (Fig. 3a,b) while presenting clear contrast in heatmap and eigengene scores when comparing ASD with control samples, indicating clear dysregulation in these processes. Interestingly, these processes were also enriched for in M3^d35^ and M22^d35^ modules.

As to modules correlated with ASD at 135 DIV, the M20^d135^ module was enriched with functional pathways related to axon guidance, epithelial adherens junction signaling, estrogen receptor signaling, PTEN signaling and NOTCH signaling pathways. The M7^d135^ module was enriched in actin cytoskeleton signaling (fibrosis), phosphoinositide metabolism pathways (Suppl. Data 5). As for GO analysis, only eight 135 DIV WGCNA modules correlated with ASD presented enrichment in GO biological processes (Suppl. Data 7). Of note, the M2^d135^ showed enrichment in pathways pertaining to neuronal differentiation and cell fate commitment, RNA metabolism, regulation of transcription, axonogenesis and proliferation processes (Fig. 3c,d) while presenting clear contrast in gene expression levels and eigengene score when comparing ASD with control samples, possibly suggesting impairment in these processes. In addition, these processes were also represented in the 135 DIV M3^d135^, M25^d135^, and M14^d135^ modules.

### Synaptic activity is dysregulated in ASD neurons

Transcriptomic analysis of ASD neurons indicated significant dysregulation of molecular mechanisms involving neuronal migration and synaptic function at an early point in their cellular development (i.e. 35 DIV). In particular, we found evidence for disturbance in pathways linked to axonal guidance and signaling, integrin signaling, regulation of actin based motility by Rho, and actin cytoskeleton signaling − all of which play a role in modulating synapse function in cortical neurons^43–48^. Because synapse function modulates overall neural network activity^49^, we first used multi-electrode array (MEA) recordings to assess the overall network activity of our cohort at the earliest differentiated time point, 35 DIV.

**Figure 4:**
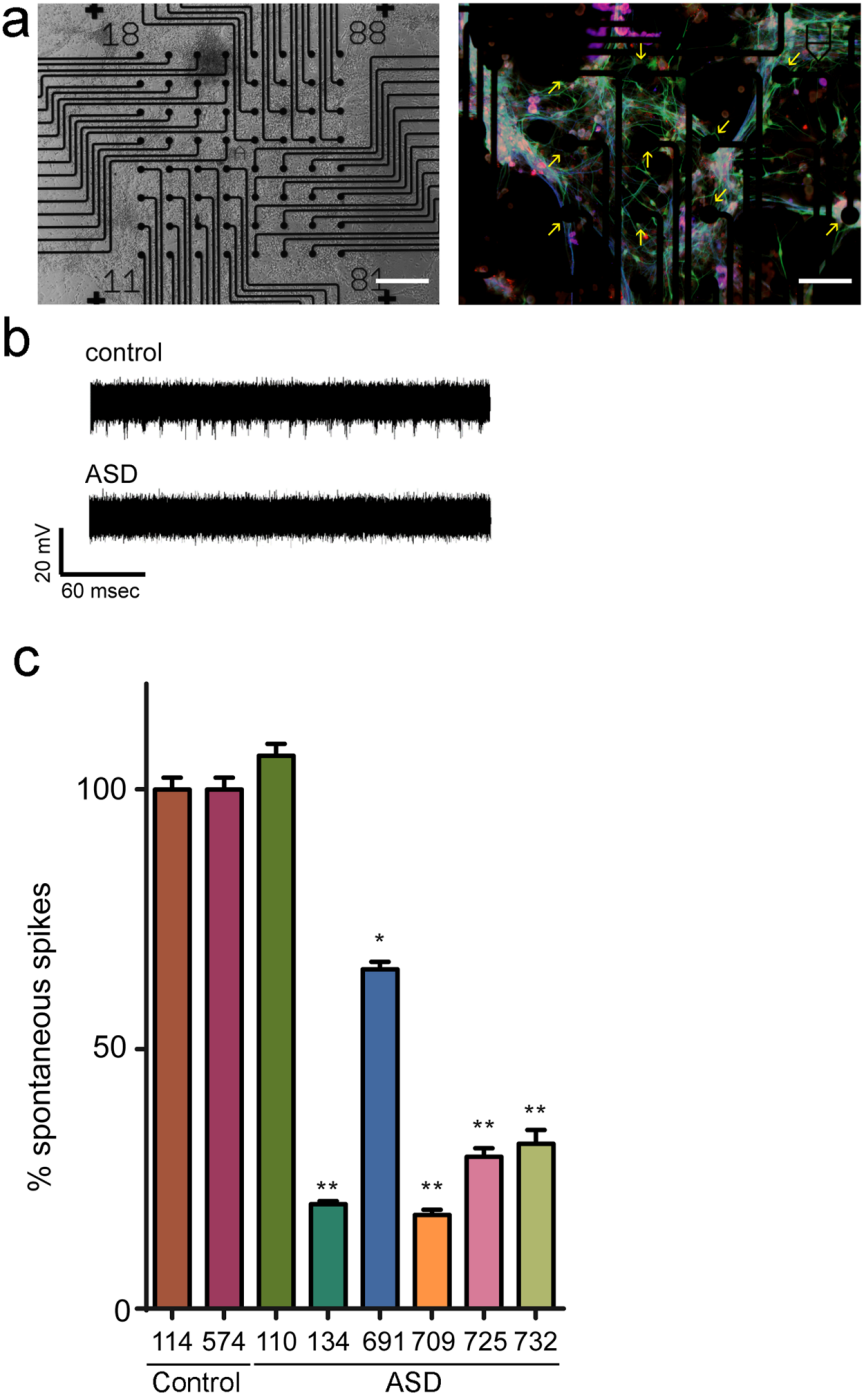
Multi-electrode array recordings reveal decreased spontaneous spiking activity in ASD iPSC-derived neurons. (**a**) Representative images (low power brightfield, right) of a well within a MEA plate. Staining for postmitotic neuronal markers MAP2 (green) NeuN (Red) and VGLUT (Blue) are observed. (**b**) Representative raw traces from an MEA recording electrode showing decreased spike activity in ASD line. (**c**) Group data at 30 DIV demonstrates that network activity is suppressed in all ASD lines except for line 110 as compared to controls (N = 16 wells, three independent replicates per time point/iPSC line, *p < 0.05; **p < 0.01 ANOVA with Tukey’s post-hoc; scale bar: 100 µM).

Spontaneous neural spiking activity was measured using the MEA, an established and robust cell based assay for synaptic function in iPSC-derived neurons^50,51^. We observed a significantly decreased level of spike activity in neural networks in all but one line (110) derived from individuals with ASD (Fig. 4). Overall spike activity was decreased by at least 50% in the remaining lines (N = 16 wells, three independent replicates per cell line for each time point; p < 0.01 ANOVA with Tukey’s post-hoc). This type of decreased MEA-measured network activity in iPSC-derived neurons from individuals with autism has been observed in other cohorts^23^.

### Calcium signaling is disrupted in ASD neurons

Coupled with the effect of spontaneous network spiking activity, calcium activity plays a central role in development^52^. Additionally, all of the neural-centric pathways that were detected by the IPA analysis are modulated in part by the activity of intracellular calcium. The type of spontaneous activity observed in iPSC-derived neurons recorded on the MEA is analogous to multiples of action potentials. These action potentials are correlated with network-wide calcium transients, resulting in the development of mature synapses^53,54^. In particular, increases in intracellular calcium as a result of spontaneous activity is implicated in the developmental expression of AMPA and NMDA receptors and a decrease in the number of silent synapses^55^.

**Figure 5:**
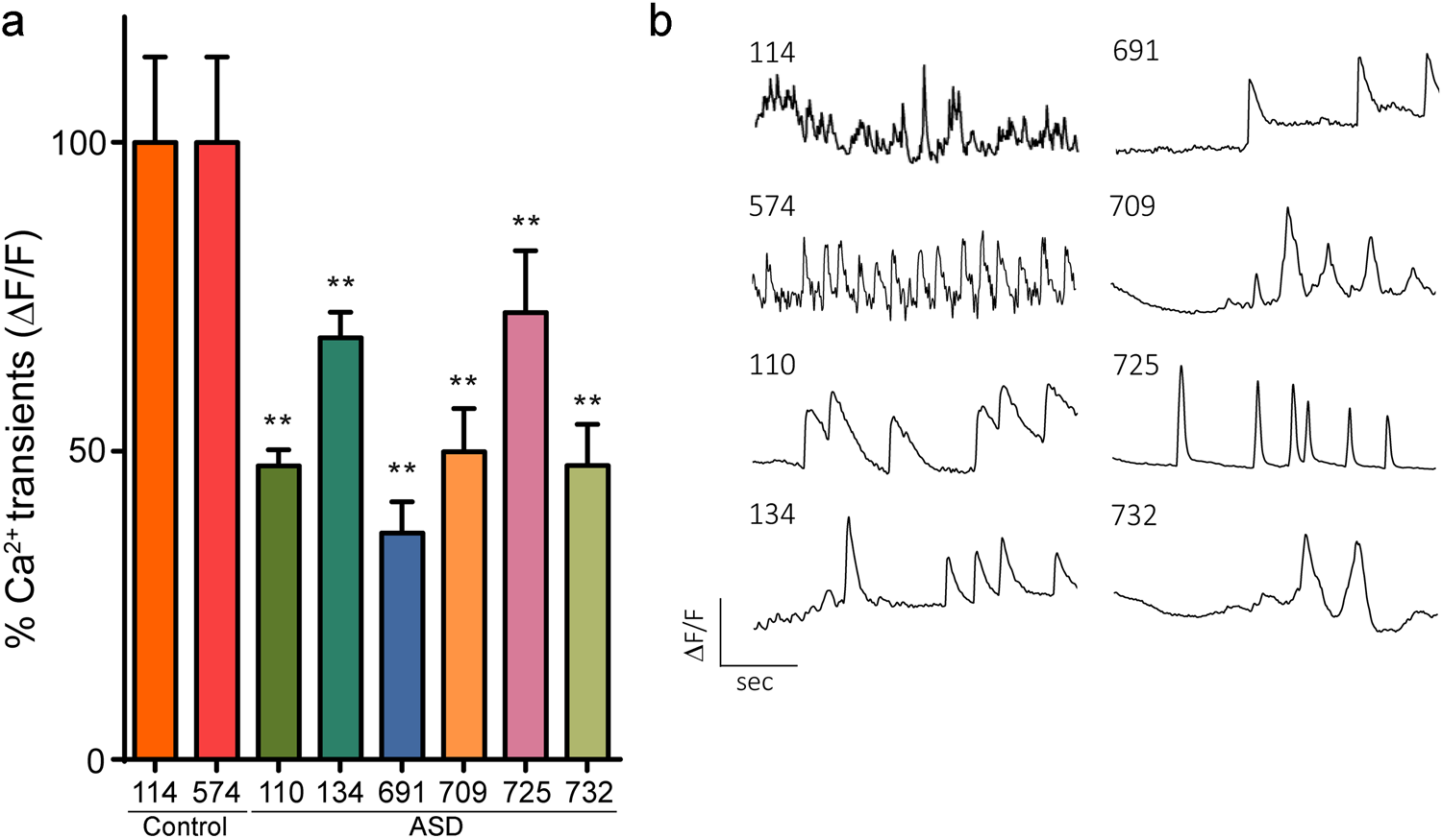
The number of spontaneous calcium transients are significantly lower in ASD iPSC-derived neurons. (**a**) Group data of Fluo-4 indicated calcium transients recorded from iPSC-derived neurons at 30 DIV are decreased. (**b**) Representative single-cell traces recorded from iPSC-derived neurons (N = 7, 40 cells across 4 ROI per well/iPSC line, 3 independent replicates; p < 0.01; ANOVA, with Tukey’s post-hoc).

In iPSC-derived neurons, intracellular calcium reflects action potential frequency, and is a readout for overall neuronal activity^56^. Moreover, calcium signaling was significantly presented as a dysregulated pathway in the M3^d35^ module of our 35 DIV WGCNA analysis (Suppl. Data 4). To complement our MEA recordings, overall spontaneous calcium transients in iPSC-derived neurons at 35 DIV were measured. A significant decrease in the number of spontaneous transients were observed at this time point in lines derived from individuals with ASD compared to controls (N = 740 cells across 4 ROI/well/line, 3 independent replicates; p < 0.01; ANOVA, with Tukey’s post-hoc) (Fig. 5). Interestingly, for line 110, MEA activity was not significantly different than controls at 35 DIV, but Ca^2+^ transients were significantly decreased. This suggests, that at least for this line, there may be some compensatory mechanisms at the synaptic level that overcome aberrant somatic Ca^2+^ signaling.

### Cell migration is dysregulated in ASD neurons

**Figure 6:**
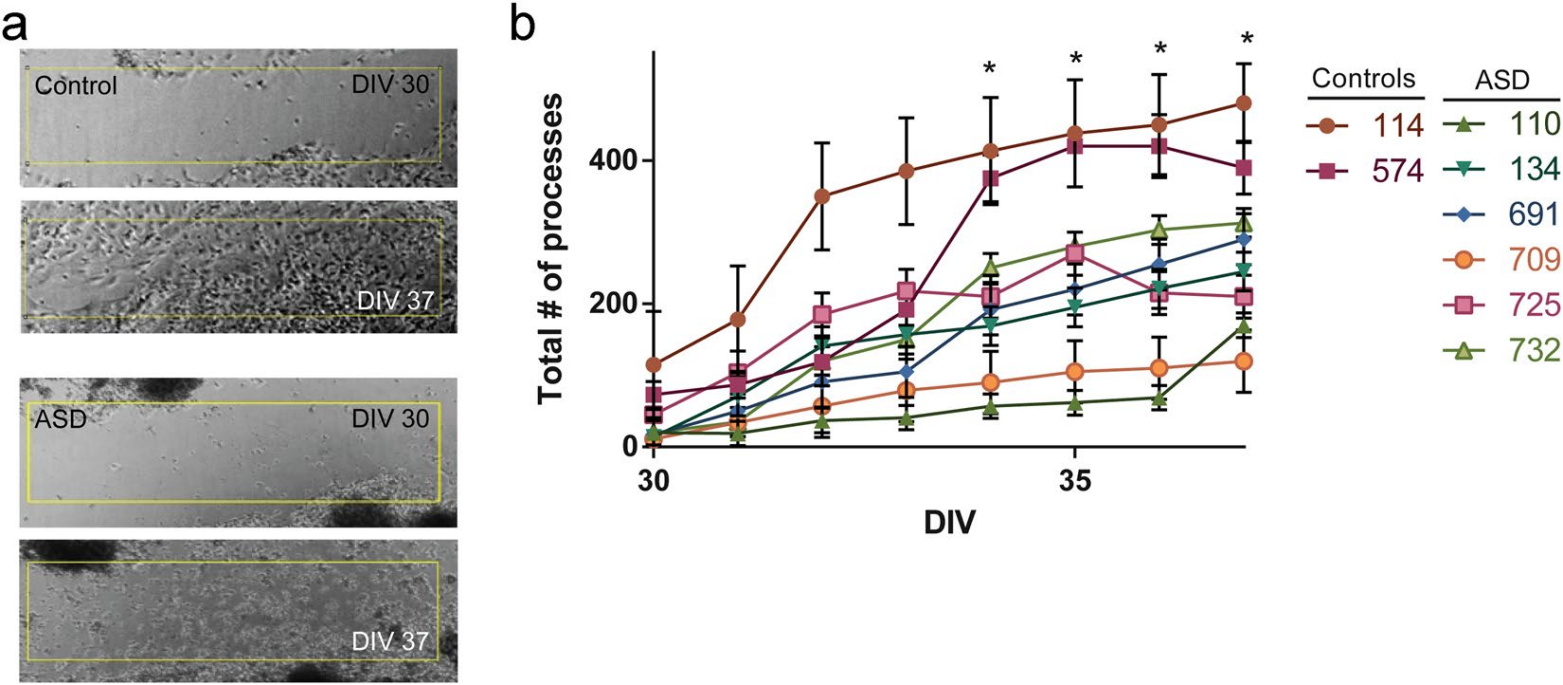
The migration of neuronal processes are significantly decreased in ASD iPSC-derived neurons. (**a**) Representative image of scratch assay. (**b**) There are significantly less neuronal processes that migrate into the scratch region over time as compared to controls. (N = 400 cells per ROI/biological triplicates; p < 0.05, Repeated Measures ANOVA).

The *in vitro* scratch assay is a cell-based assay for neuronal and axonal migration studies^57,58^. Therefore, we used a standard single-scratch assay to measure the amount and extent of neuronal process migration at 35 DIV in our cohort. We observed a decrease in the number of processes that reinvaded the scratch area (N = 400 cells/ROI; p < 0.01, Repeated Measures ANOVA; Fig. 6). Of note, it was not until after the 4^th^ day of imaging that a significant difference between the cases and the controls emerged. One possibility for this delay is that we did not employ the use of a chemoattractant combined with a scratch. Additionally, the cells at the scratch edge lose their original morphology, a phenotype that implies a loss in the ability of the cells to recover and acts as a physical block to invading cells^59,60^. Nonetheless, the lines in our autism cohort showed significantly fewer processes within the scratch area, a finding reported by other groups for neurons derived from iPSCs from individuals with ASD^23^.

## Discussion

Studies to date have pointed to numerous mechanisms underlying ASD. Nonetheless, the uniqueness of our present study is threefold. The first is that it employs a model that consists of idiopathic ASD-specific iPSCs differentiated into cortical neurons, thereby generating a largely inaccessible cell type directly from ASD patients. The second aspect is the ability to track transcriptional changes along the neuronal differentiation process of ASD and control cell lines, which mirrors the changes occurring during human development. The third distinct feature is the use of multiple analytical approaches to investigate at two distinct time points significant transcriptional changes.

The iPSC lines used in this study were successfully developed from PBMCs obtained from six individuals with idiopathic ASD and two unaffected individuals, as well as three commercially available lines. These iPSCs expressed a panel of pluripotency markers (Suppl. Figure 1). Furthermore, the genomic stability of the of iPSC lines was demonstrated through karyotype analysis (Suppl. Figure 2) and by validating the preservation of previously identified mutations (Suppl. Figure 3), which may contribute to ASD risk^28,29^.

The whole-transcriptome expression patterns of patient and control iPSC-derived neurons was examined through a neuronal differentiation time course spanning 135 days of differentiation. The data show that ASD-related transcriptional disturbances exist in neuronal cultures shortly after the induction of terminal differentiation on day 35 as compared to cultures that had been differentiating for an additional 100 days, suggesting that a disparity exists in the developmental trajectory of ASD neurons. Analyses by IPA and GO revealed a significant enrichment of genes involved in biological processes previously implicated in autism, including synaptic function, axon path-finding, cell fate specification, and activity-dependent regulation of gene expression, such as the Sonic Hedgehog (SHH)/GLI1 signaling pathway. In fact, SHH has been previously implicated in ASD^61,62^, cell fate^63^, axon guidance^64^, patterning and regionalization^65^. Analysis of the DEGs at 35 DIV and 135 DIV against a curated set of gene expressed in 1) iPSC derived neurons or (2) BrainSpan gene expression data for the developing fetal brain showed an overrepresentation of similar GO categories to those seen with the whole transcriptome analyses. These result highlight the importance of genes involved in forebrain development, axonogenesis and axon guidance, and cell migration as differentially expressed between ASD patient-derived neurons and the control neurons. These themes were also recapitulated by WGCNA analysis. Hence, an unbiased characterization of the ASD patient-derived cortical neurons indicated a dysregulation of critical genes that orchestrate the development of specific brain regions and spatiotemporal regulation of neuronal cell fate acquisition.

Moreover, the WGCNA analyses showed consistent and significant altered expression of molecular mechanisms involving the structure and signaling interactions of the cytoskeletal matrix. Specifically, we observed a deregulation of genes involving matrix structure, specifically genes coding for collagen fibrils, actin fibrils, actin polymerizing factors and POTEF, that are critical for morphogenesis and axon guidance. Furthermore, WGCNA of individual time points identified modules of highly co-expressed genes that were significantly correlated with the expression patterns of ASD-derived neurons. Many of the modules correlated with ASD could be straightforwardly characterized by IPA and GO with significant enrichment in categories related to biological functions that are crucial for brain development.

Of note, certain pathways in our IPA and GO analyses did not, at first glance, directly correlate with known neuronal or brain developmental functions. Nonetheless, a closer look at the genes driving the significance of those pathways showed that their functions overlapped with many of the pathways mentioned above. For instance, the “hepatic stellate cell activation” pathway in our 35 DIV time point IPA analysis (Fig. 2b) include many of collagen and myosinfibrils genes (*COL1A1,COL8A1, COL5A2, COL15A1,COL3A1, COL4A5, COL24A1, COL21A1, COL11A1, COL12A1, COL4A6, MYH9,MYL9*), suggesting misregulation of the cytoskeletal matrix signaling pathway. In another instance, the “cardiomyocyte differentiation via BMP receptors” pathway in our 35 DIV time point IPA analysis (Fig. 2b) include many of same BMP genes found in the “axon guidance signaling” pathway demonstrating that many of the genes driving seemingly unrelated pathways overlap with genes in pathways critical for neurodevelopment.

Given the clinical and genetic heterogeneity of ASD and the individual-to-individual variance in the human population, it was not surprising that not every sample in each group followed exactly the same pattern of gene expression when specific gene ontologies and molecular functions were analyzed. For example, in Fig. 3a, two of the control samples had a pattern similar to the ASD samples in module 13 (day 35). However, the overall analysis of the cases and controls showed a statistically significant difference between the groups. In the examination of additional modules, these two samples fall in line with the pattern of the other controls (For example, module 2 day 135, Fig. 3b). In fact, it is remarkable that despite the intrinsic heterogeneity between human samples, we were still able to identify statistically significant differences between the ASD samples and the controls.

In our effort to verify the physiological relevance of our RNA-Seq analyses, we used MEA, calcium transients electrophysiology, and the scratch assay to probe for functional disturbances in some of the most recurrent biological pathways that were presented at 35 DIV, including synaptic activity, calcium signaling, and cell migration. Thus, we used MEA to examine spontaneous synaptic activity and detected a significant decrease in neurons derived from ASD individuals. This decrease is key because spontaneous neuronal activity plays an important role during the development of neural circuits in the brain^66^. Specifically, spontaneous network-level spiking drives the strength of GABAergic and glutamatergic synapses early in development and may play a role in regulating the hypothesized imbalance in excitatory and inhibitory inputs in autism^67–69^. Our MEA results paralleled our calcium transients and cell migration results, showing an overall decrease in the neuronal activity in most ASD lines at 35 DIV. In addition to the role calcium plays in overall network activity, spontaneous changes in intracellular calcium transients have been linked to actin cytoskeletal rearrangement and modulation of the activity of integrins, two pathways implicated at this time point via our IPA analysis^70^. Hence, these results support the WGCNA analysis which implicated a dysregulation of calcium signaling and cell migration. There is a complex interplay between synaptic activity, calcium activity and neuronal migration in development^52,71,72^. Most of the neuron-specific IPA pathways identified at DIV 35 could be linked to the migration of neurons. Not surprisingly, the migration of neurons has been implicated in autism and the disruption of minicolumns in the cortex^73,74^. Combined, our cell-based observations compellingly support our transcriptomic analyses at DIV 35.

Another interesting dysregulated biological process that our study presents, for the first time, as an underlying mechanism for ASD is DNA and RNA metabolism. For instance, IPA analysis of differentially expressed genes at 135 DIV, which highlighted Guanosine nucleotide metabolism as a significantly altered pathway, presented significantly differentially expressed genes coding for enzymes like xanthine dehydrogenase and guanine deaminase (*XDH* and *GDA*).

It should be emphasized that differential expression analysis and WGCNA are distinct methodological approaches to identifying biological relationships between altered patterns of genes expression and disease. Thus, the agreement in the results from these different analytical approaches provides greater support for the biological relevance of the data.

The emergent, overall picture is that convergent molecular disturbances in ASD impact synaptic development and function, metabolism, and cellular molecular interactions involving the cytoskeletal matrix (i.e. axon guidance and cell migration). It is still unknown how the interaction between various disturbed mechanisms in this cohort of ASD-derived neurons leads to these transcriptional profiles and cellular phenotypes in the patients’ cells; this warrants further investigation in future studies.

## Methods

### Ascertainment and clinical criteria

Informed consent was obtained from all study participants under a University of Miami Miller School of Medicine Institutional Review Board approved protocol. ASD individuals used for generation of iPSCs (Table 1) were ascertained following an ASD diagnosis. Core inclusion criteria for this study were as follows: (1) between 3 and 21 years of age, (2) a presumptive clinical diagnosis of ASD, (3) an expert clinical determination of an ASD diagnosis using DSM-IV criteria^75^ supported by the Autism Diagnostic Interview-Revised (ADI-R)^76^, and (4) an IQ equivalent >35 or developmental level >18 months as determined by the Vineland Adaptive Behavior Scale (VABS)^77^. Diagnostic determination was based on review by a panel consisting of experienced clinical psychologists and a pediatric medical geneticist. IQ was obtained for the majority of individuals from administration of any of several measures (for example, age appropriate Wechsler scale, Leiter intelligence test, or Mullen Scales of Early Learning, MSEL) or from medical records. All individuals were enrolled under a University of Miami IRB approved protocol and all experiments using iPSCs were performed in compliance with the guidelines and regulations of the institutional biosafety committee at the University of Miami. The control iPSC lines that were used in this analysis came from different sources. Two of the samples that were used were reprogrammed from healthy, cognitively normal control NHW males that were over 18 years of age. The reprogramming was performed in the same manner as the ASD cases (see below) (Cont114 and Cont574). Cont1021 was obtained from the ATCC as an iPSC clone derived by Sendai reprogramming from cardiac fibroblasts obtained from a NHW male (ATCC-CYS0105). The two additional control iPSCs (Cont101 and Cont102) were obtained from the reprogramming of human foreskin fibroblasts from NHW male individuals (System Biosciences). The pluripotency of all controls was verified by immunocytochemistry (see below).

### Isolation and culture of peripheral blood mononuclear cells

Peripheral blood was isolated from six ASD-affected males (Table 1) following informed consent under University of Miami guidelines and regulations. In addition, iPSC lines were derived from peripheral blood from two unaffected control male individuals. Additional control iPSC derived from fibroblasts were obtained from the American Type Culture Collection (ATCC) (ACS 1021 and ACS 1011) and Systems Biosciences (SBI1). Ficoll-Hypaque (GE Healthcare Life Sciences) density-gradient centrifugation was used to isolate mononuclear cells from peripheral blood. Isolated peripheral blood mononuclear cells (PBMCs) were cryopreserved in CryoStor10 freezing medium (STEMCELL Technologies). For four days prior to infection, PBMCs were cultured in suspension at a density of 5 × 10^5^ cells/mL in StemPro®−34 Medium (PBMC medium; STEMCELL Technologies) supplemented with 100 ng/mL SCF, 100 ng/mL FLT-3, 20 ng/mL TPO, and 10 ng/mL IL-6 (all Peprotech). During the four days prior to transduction up until day 8 post-transduction, half of the spent media was exchanged for fresh media every other day. All cultures described here were kept in a 37 °C incubator with 5% CO_2_.

### Reprogramming of PBMCs into pluripotent stem cells

Transductions were carried out on 1–5 × 10^5^ cells/well in a 24-well plate using Oct4, Sox2, Klf4, and c-Myc Cytotune™ Sendai viruses (Life Technologies) at a MOI of 3–6 in the presence of 4 μg/mL polybrene. Sendai viruses were removed 24 hours post-infection by centrifuging the cells at 400 × g for 10 minutes, aspirating the viral supernatant, resuspending the cells in fresh PBMC medium, and then transferring them back to the in the 24-well plate. A small volume of fresh media was immediately added to the wells after the cells were collected for centrifugation to prevent the desiccation of cells which might have remained in the well. On day 3 post-transduction, 2 × 10^4^ and 1 × 10^5^ PBMCs were seeded into 60 mm mitomycin-c treated primary mouse embryonic fibroblast (MEF; Millipore) feeder layer culture dishes in PBMC medium. At this time, SCF, FLT-3, TPO, and IL-6 were omitted from the culture medium and we began supplementation with 10 µM CHIR99021, 1 µM PD325901, 1 µM thiazovivin, and 10 µM Y27632 (Stemgent) which were continually added to the culture medium thereafter. On day 7, the cells were transitioned to iPSC culture medium by introducing mTeSR1 to the PBMC medium at a 1:1 ratio. The following day, the culture medium was fully switched over to mTeSR1 with the media being changed daily thereafter. Transduced cell cultures were visually examined every day for the appearance of proliferating cell clusters, indicative of cells undergoing reprogramming. Candidate iPSC colonies showing ESC morphology were manually collected, expanded and analyzed for pluripotency.

### Validation of genomic stability in iPSC lines

The karyotypes of the blood-derived iPSC cultures were analyzed via G-banding. Validation of genetic variant preservation in ASD patient-specific iPSC lines was carried out by first isolating genomic DNA using a DNeasy Blood & Tissue Kit (Qiagen) and performing Sanger sequencing. Primers were created with the Primer3 program and the sequencing reaction was run with the Big Dye Terminator v3.1 on an Applied Biosystems 3130xl DNA Analyzer (Life Technologies). Results were evaluated in the Sequencher v4.10.1 program (Gene Codes Coorporation, Suppl. Figures 2 and 3).

### Differentiation of iPSCs into cortical progenitors and neurons

All of the neurons, both ASD and control, were differentiated from iPSC lines using the following protocol. Prior to inducing neural differentiation, iPSCs were isolated from MEF feeder layer cells through magnetic column separation using MEF-specific antibodies (anti-mEF-SK4) coupled to paramagnetic beads (Miltenyi Biotec). Magnetic separation of the cells was carried out according to the manufacturer’s instructions with few exceptions. Briefly, iPSC colonies were dissociated into a single cell suspension through a 10-minute treatment with Accutase. In lieu of MACS buffer, mTeSR1 containing 2 µM thiazovivin and 20 µM Y27632 was used for both incubation of the cells in MEF-specific antibodies and for column washes during magnetic separation.

Cortical neuron differentiation of the iPSCs was performed by providing recombinant growth factors and small molecule compounds in the growth media in a specific schedule (Suppl. Figure 4A and Suppl. Table 1). Additionally, the diagram shown in Suppl. Figure 4A illustrates the step-wise procedure used for cortical neuron differentiation. Cortical neurogenesis was initiated through the formation of neural EBs using AggreWell™800 plates (STEMCELLTechnologies) according to the manufacturer’s protocol. Briefly, a single cell suspension of 3–4.5 × 10^6^ iPSCs (10,000 to 15,000 cells per neural aggregate) was dispensed into each well of an Aggrewell™800 plate in neural induction media (NIM; STEMCELL technologies) supplemented with 10 μM Y27632, 10 μM SB431542 (Stemgent), 1 μM dorsomorphin (Stemgent), and 1 μM thiazovivin.

After five days, neural aggregates were collected from the AggreWell™800 plate and transferred to 6-well plates coated with 15 μg/mL Poly-L-Ornithine (Sigma) and 10 μg/mL laminin (Trevigen) and cultured for another five days in NIM. On day 12, the differentiation medium was transitioned from NIM to an N2/B27 enriched neurobasal medium (ENB) consisting of a 1:1 mixture of DMEM/F12 (with L-Glutamine) and Neurobasal™ medium (minus phenol red; GIBCO), 0.5% N2® Supplement (GIBCO), 1% B-27® Supplement (GIBCO), 0.5% non-essential amino acids, 0.5% GlutaMAX (GIBCO), 1% Insulin-Transferrin-Selenium-A (GIBCO), 1% Antibiotic-Antimycotic, 30 ng/mL tri-iodothyronine (Sigma), 40 ng/mL thyroxine (Sigma), 100 µg/ml bovine-serum albumen (Sigma), 60 ng/ml progesterone (Sigma), 16 µg/mL putrescine(Sigma)^78^, 5 μg/mL N-acetyl-L-cysteine (Sigma), and 5 μM foreskolin (Sigma). Between days 12–35, cortical progenitor cells were expanded by enzymatically passaging the cells with Accutase or Accumax (Sigma) every 4–7 days and replated at a density of 4–8 × 10^4^ cells/cm^2^. On day 35, the cells were plated for terminal differentiation on culture plates coated with 100 μg/mL Poly-D-Lysine (Trevigen), 20 μg/mL laminin, and 10 μg/mL fibronectin (Trevigen) at a density of 2–4 × 10^4^ cells/cm^2^. Of note, the seeding densities listed are based on single cell counts and do not reflect cells contained within neural aggregates, which were numerous in culture.

### Immunocytochemistry and fluorescence imaging

For immunocytochemistry (ICC) and imaging, cultures were fixed with 4% formaldehyde (Thermo Scientific Pierce)/4% sucrose (Sigma) for 15 minutes at room temperature and washed with PBS. The slides were simultaneously blocked and permeabilized for 45 minutes at room temperature in 20% normal donkey serum (NDS) or normal goat serum (NGS) (both from Jackson ImmunoResearch) and 0.2% Triton X-100 (Sigma) in a modified antibody buffer (150 mM NaCl, 50 mM Trizma base, 1% BSA, 100 mM L-Lysine, 0.04% sodium azide, pH 7.4, 0.3 M glycine; Sigma) (Blackmore *et al*., 2012). Suppl. Table 2 lists all of the primary antibodies used for ICC analysis. Cultures were incubated in primary antibody solutions overnight at 4 °C then washed extensively in ICC wash buffer (PBS/1% BSA) and incubated with the appropriate Alexa-fluor conjugated secondary antibody (Life Technologies) for 1 h at RT. After the incubation in the secondary antibody, the cells were washed extensively in ICC wash buffer and incubated with DAPI (NucBlue Fixed Cell Stain; Life Technologies). Images were either acquired using a Nikon Eclipse TE2000U fluorescence inverted microscope or a Zeiss LSM 710 confocal microscope (Carl Zeiss Microscopy). Cells imaged with a confocal microscope were grown in either 4-well or 8-well chamber-well slides (Millicell EZ SLIDES; Millipore) coated with coated with 100 μg/mL Poly-D-Lysine, 20 μg/mL laminin, and 10 μg/mL fibronectin (PDL/L/F; Trevigen). After secondary staining, the chambers were removed and the slides were mounted with glass coverslips using DAKO Fluorescence Mounting Medium (DAKO).

### Purification of total RNA

Total RNA was extracted was extracted from cells using a PureLink RNA Mini Kit (Ambion) using the manufacturer’s protocol with several modifications. Cell lysates were homogenized using QIAshredder spin columns (Qiagen). Two distinct methods were used to remove genomic DNA during the RNA purification: (1) homogenized lysates were filtered through a gDNA eliminator spin columns (Qiagen) and (2) on-column DNase treatment (Qiagen). The chemistry behind silica-membrane spin column-based RNA purification necessitates ethanol concentrations ≥50% (final concentration) during precipitation and wash steps in order to obtain RNA from approximately 18 nucleotides (nt) upwards. Thus, to extract all species of RNA, including those <200 nt such as miRNA, after homogenized lysates were passed through gDNA eliminator spin columns, 100% ethanol (Sigma) was used to precipitate RNA out of solution (final ethanol concentration of 50%). To this end, we additionally used Buffer RWT (Qiagen) in place of PureLink RNA Mini Kit Wash Buffer I. Finally, RNA was eluted in the Ambion® RNA Storage Solution. The concentration and quality of RNA samples was assessed using a Bioanalyzer RNA kit (Agilent Technologies) to determine the RNA integrity number (RIN).

### Whole transcriptome sequencing

For each sample, ≥750 ng of total RNA (RIN between 7.9 to 10) was processed for RNA-Seq. Ribosomal RNA was depleted using the Ribo-Zero rRNA Removal Kit (Epicentre) and cDNA libraries were generated with ScriptSeq mRNA-Seq Library Preparation Kit (Epicentre), which enables directional sequencing through stand-specific tagging. Paired-end sequencing was performed on indexed samples and run on the HiSeq. 2500, yielding a minimum of 50 million reads/sample. Alignment of RNA-Seq reads against the human genome (GRCh38) was performed using STAR Aligner and Htseq-count was used to count the number of overlapping reads with genes. Differential expression analysis was conducted using EdgeR^79^. An FDR cut-off of 0.05 was used for all of the tests.

### Hierarchical clustering

Hierarchical clustering dendrograms were constructed using simple R functions. Log_2_CPM values were extracted for all the differentially expressed genes and formatted for all samples in the day 35 and day 135 time points. A dissimilarity matrix was calculated for each time point using the Euclidan method within the dist() function. The distances calculated from the dist() function was fed into a hierarchical clustering function hclust() using the Ward.D2 method. The dendrograms were produced using the plot function.

### Weighted gene co-expression network analysis (WGCNA)

RNA-Seq data from the two time points was analyzed with weighted gene co-expression network analysis through the R package WGCNA^80^. Genes with exorbitant numbers of missing samples were filtered using the function goodSamplesGenes. For network construction and module detection, the blockwiseModule function was used with the following parameters: maxBlockSize = 10000, corType = “bicor”, maxPOutliers = 0.1, pearsonFallback = “individual”, networkType = “signed”, replaceMissingAdjacencies = TRUE, deepSplit = 2, detectCutHeight = 0.995, minModuleSize = 30, pamStage = FALSE, reassignThreshold = 1e^−6^, mergeCutHeight = 0.15, verbose = 3, checkMissingData = TRUE, and numericLabels = TRUE. Next, modules that were significantly associated with the group of ASD individuals were identified by correlating the module eigengene profiles with individuals manifesting a clinically diagnosed ‘ASD phenotype’.

### Ingenuity pathway analysis (IPA)

The “Core Analysis’ function included in QIAGEN’s Ingenuity® Pathway Analysis (IPA®, QIAGEN Redwood City, www.qiagen.com/ingenuity) was used to interpret the human RNA-Seq and WGCNA data in the context of biological processes, pathways and networks. After the analysis, generated networks were ordered by p value significance. This pathway analysis tool generates networks where the differentially regulated genes can be related according to previously known associations between genes or proteins.

### Go ontology (GO) analysis

RNA-Seq and WGCNA data generated were also analyzed using the Biological Networks Gene Ontology tool (BiNGO). BiNGO is an open-source Java tool to determine which Gene Ontology (GO) terms are significantly overrepresented in a set of genes. We used BiNGO version 3.0.3 which was released on April 7^th^ 2015 and uses ontology annotations from the Gene Ontology Consortium. BiNGO mapped the predominant functional themes of the tested gene set on the GO hierarchy^37^. Enrichment of GO biological processes was initially assessed relative to a human genome background set. Enrichment of GO biological processes was additionally assessed in DEG lists relative to two neuronal background sets. The first neuronal background set is the subset of protein-coding genes expressed across all neuronal lines for both time points. The second neuronal background set is a list of all genes expressed across RNA-Seq human fetal brain samples from the BrainSpan Atlas (www.brainspan.org)^38^.

### Multi-electrode array (MEA)

After differentiation to 30 days, neurons were plated in 6-wells of a 12-well MEA plate (Axion Biosystems) and recorded after recovery for five days. Each well contains an 8 × 8 grid of 30 nm circular nanoporous platinum electrodes embedded in the cell culture substrate, with a pole-to-pole electrode spacing of 200 μM. Each well was treated with 0.1% polyethylenimine (PEI) in sodium borate buffer, pH 8.4. After treatment, each well was coated in laminin (6 μg/mL) and neurons were plated at 150,000 neurons/well dotted on the electrode grid^51^. Cells were then fed every other day using standard culture media. Extracellular recordings of spontaneous action potentials were performed in culture medium at 37 °C using a Maestro MEA system and AxIS software (Axion Biosystems). Data were sampled at a rate of 12.5 kHz with a hardware frequency bandwidth of 200–5000 Hz, and filtered again in software using a 200–2500 Hz single-order Butterworth band-pass filer to remove high frequency noise before spike detection. The threshold for spike detection was set to 5.25 times the rolling standard deviation of the filtered field potential on each electrode. Ten-minute recordings were used to calculate average spike rate for the well. Spike time stamps were exported to Neuroexplorer (NEX Technologies) for creation of spike raster plots. Lines were recorded as independent biological triplicates.

### Calcium imaging

iPSC lines that had been neuronally differentiated for 35 days were pretreated with an HBSS loading buffer containing 20 mM HEPES, 2.5 mM probenecid, and Fluo-4NW (Life Technologies) for 30 min at 37 °C and then at room temperature for an additional 15 min before imaging. Cells imaged were plated and treated under the same conditions as cells used for electrophysiological experiments. Cells were imaged at room temperature using a Zeiss LSM 780 inverted spinning disc confocal microscope and Zeiss Blue software. Time-lapse images were taken every one second for ten minutes using 512 × 512 pixel resolution and cells were excited using the 488 nm laser line. Regions of interest (ROIs, 5 µm × 4 µm) were drawn over each cell soma and any change in fluorescence intensity over time was calculated within the imaging software. Within a given imaging field, only active cells were chosen for analysis^56^. All images were background subtracted and the analysed with a custom written routine in Matlab (Mathworks, Natick, MA) based on the “PeakFinder” algorithm^81^. In each signal trace, the routine calculates the area of the signal with vertices being the maximum itself and the furthest monotonically decreasing minima on either side. This is used to model the relevance of each signal peak. Relevant peaks are defined as maxima that have an associated area greater than one standard deviation from the mean of the signal. These peaks are then used in calculation of event frequencies. Five imaging fields were chosen per well across 12 wells per condition and across three independent biological replicates, which averaged 246.6 cells per replicate per line.

### Scratch assay

After 23 days in culture, cells were harvested with 1 mL pre-warmed Accutase (Sigma) and pelleted at 129xg for five minutes followed by cell count determined on a Countess II FL (Life Technologies). NSCs were then plated onto poly-ornithine (0.01%; Sigma) and laminin (133 µg/mL in Liebowicz medium; Sigma) coated 24-well Black Wallac-Visiplates (Perkin Elmer) at 85,000 cells/well and allowed to recover for seven days. Cells were then 80–90% confluent and subject to a 0.5 mm injury along the diameter of each well using a pulled glass micropipette^59,82^. Following scratch, cultures were allowed to recover for two hours and then imaged on an EVOS FL (Life Technologies) and every 24 hours for ten days. Images were analyzed for differences in recovery from the scratch injury by rate and density of repopulation of scratched area using ImageJ (NIH). Counts were done within a standardized area of 1.3 × 0.35 mm daily on cell body and process migration. This standardized area and starting cell density are important for accurate quantification of results^59^. Experiments were performed across multiple wells per line and in biological triplicates.

### Statistical analysis

For the RNA-Seq analysis of differentially expressed genes, differential expression analysis was conducted using EdgeR^79^. An FDR cut-off of 0.05 was used. In IPA and GO analyses a permutated p value of less than 0.05 was used to choose the top 15 pathways and biological processes. In MEA, calcium imaging and scratch assay experiments, either ANOVA with Tukey’s post-hoc or repeated measures ANOVA were used. All experiments were completed as independent triplicates for each condition and all statistics are reported as ± S.E.M.

### Data availability

The whole transcriptome data analyzed in this manuscript are available through the Gene Expression Omnibus data repository.

## Electronic supplementary material

Supplemental Figures and Tables

Supplementary data file 1

Supplementary data File 2

Supplementary data file 3

Supplementary data file 4

Supplementary data file 5

Supplementary data file 6

Supplementary data file 7
