# Author Correction: Convergent Pathways in Idiopathic Autism Revealed by Time Course Transcriptomic Analysis of Patient-Derived Neurons

**DOI:** 10.1038/s41598-022-07356-4

**Published:** 2022-02-24

**Authors:** Brooke A. DeRosa, Jimmy El Hokayem, Elena Artimovich, Catherine Garcia-Serje, Andre W. Phillips, Derek Van Booven, Jonathan E. Nestor, Lily Wang, Michael L. Cuccaro, Jefery M. Vance, Margaret A. Pericak-Vance, Holly N. Cukier, Michael W. Nestor, Derek M. Dykxhoorn

**Affiliations:** 1 John P. Hussman Institute for Human Genomics, University of Miami Miller School of Medicine, Miami, FL 33136 USA; 2 Department of Medical & Molecular Genetics, Oregon Health & Science University, Portland, Oregon 97239 USA; 3 The Hussman Institute for Autism, Baltimore, MD 21229 USA; 4 John T. Macdonald Foundation Department of Human Genetics, University of Miami Miller School of Medicine, Miami, FL 33136 USA; 5 Department of Neurology, University of Miami Miller School of Medicine, Miami, FL 33136 USA; 6 Department of Public Health Sciences, University of Miami Miller School of Medicine, Miami, FL 33136 USA

Correction to: *Scientific Reports* 10.1038/s41598-018-26495-1, published online 30 May 2018

The Data Availability statement was not included in this Article. It is now included below:

**Data Availability**

Transcriptomic data generated for this study have been deposited in Short Read Archive and can be accessed using the BioProject number PRJNA511583.

