# Supplementary material for: Convergent Pathways in Idiopathic Autism Revealed by Time Course Transcriptomic Analysis of Patient-Derived Neurons
Source: Sci Rep. 2018 May 30;8:8423. doi: 10.1038/s41598-018-26495-1 (PMC5976773; doi:10.1038/s41598-018-26495-1)
Supplement: Supplementary file 1 — Supplemental Figures and Tables [file 41598_2018_26495_MOESM1_ESM.pdf]

Convergent Pathways in Idiopathic Autism Revealed by Time Course Transcriptomic Analysis of Patient-Derived Neurons

Brooke A. DeRosa, Jimmy El Hokayem, Elena Artimovich, Catherine Garcia-Serje, Andre W. Phillips, Derek Van Booven, Jonathan E. Nestor, Lily Wang, Michael L. Cuccaro, Jeffery M. Vance, Margaret A. Pericak-Vance, Holly N. Cukier, Michael W. Nestor, Derek M. Dykxhoorn

Supplementary Figures and Tables

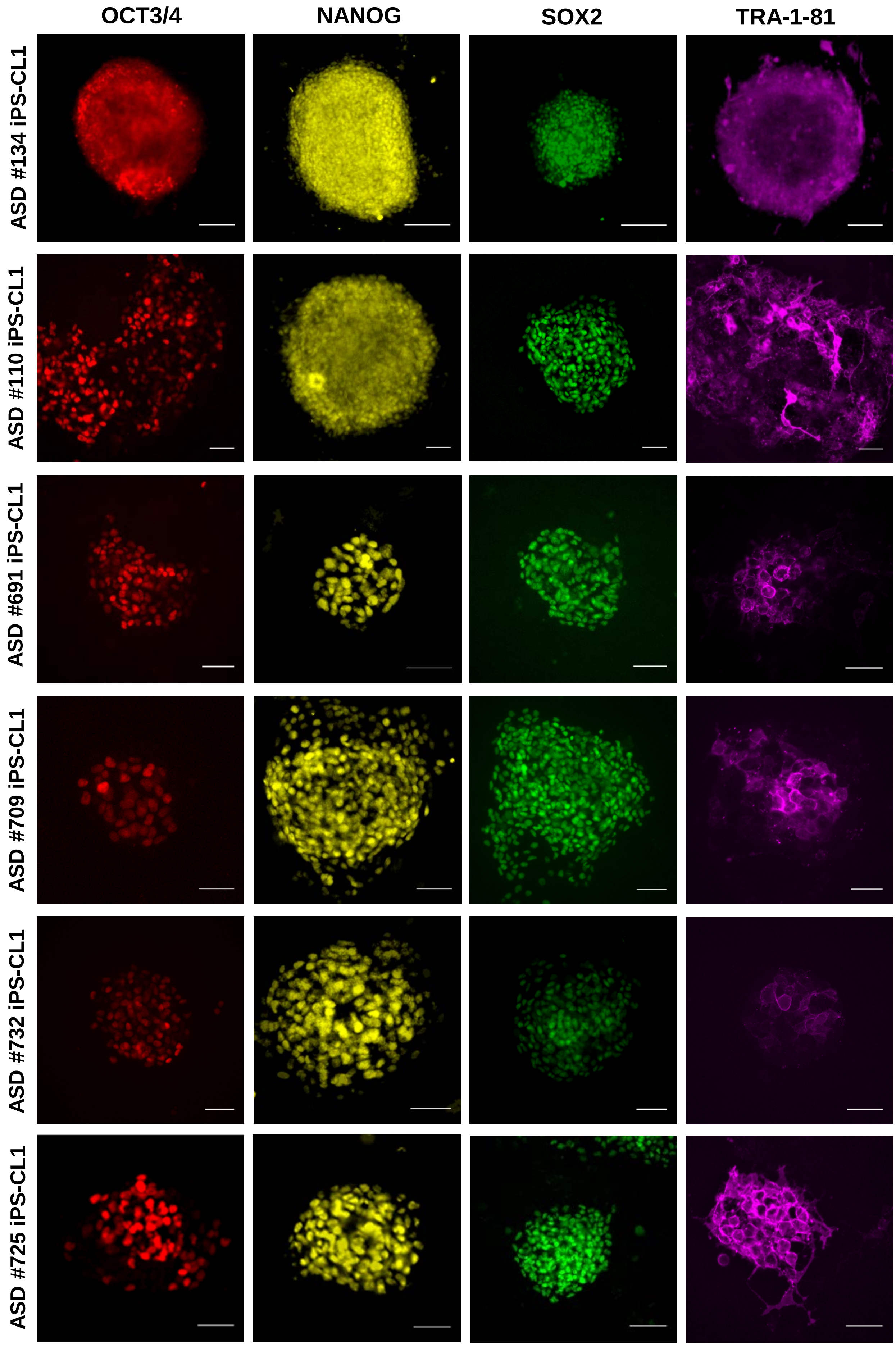

Supplemental Figure 1 | Derivation of patient-specific iPSCs from PBMCs. Representative images of immunostained colonies are shown for iPSCs derived from ASD individuals. The iPSC lines stain positive for markers of pluripotency: Oct3/4, Nanog, Sox2, TRA-1-81. Note that Oct3/4, Nanog, and Sox2 plurpotency markers are localized to the nucleus (transcription factors) whereas TRA-1-81 is a surface marker. Scale bar: 50 μm.

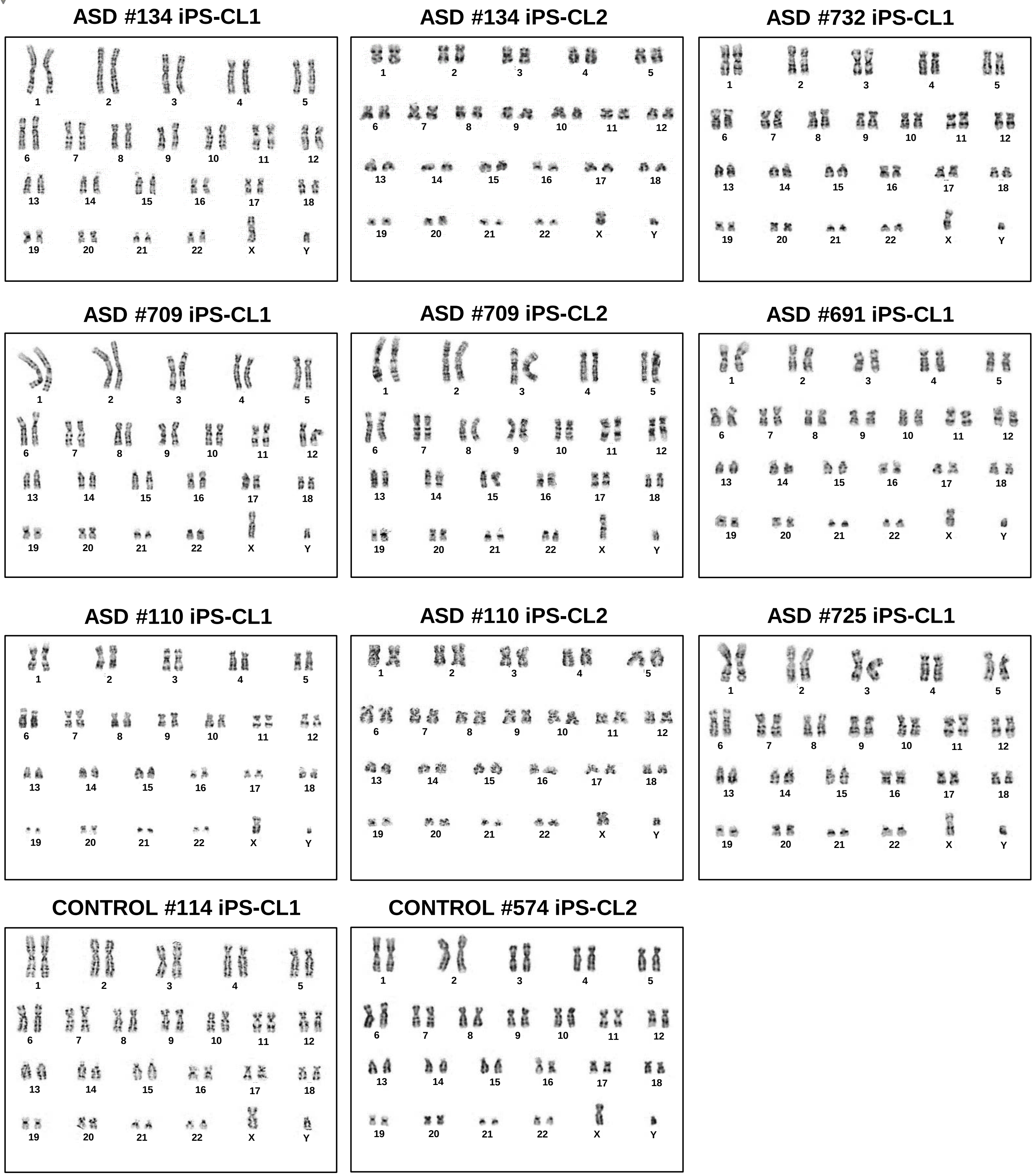

Supplemental Figure 2 | Karyotypes of non-commercial iPSC lines used in study.

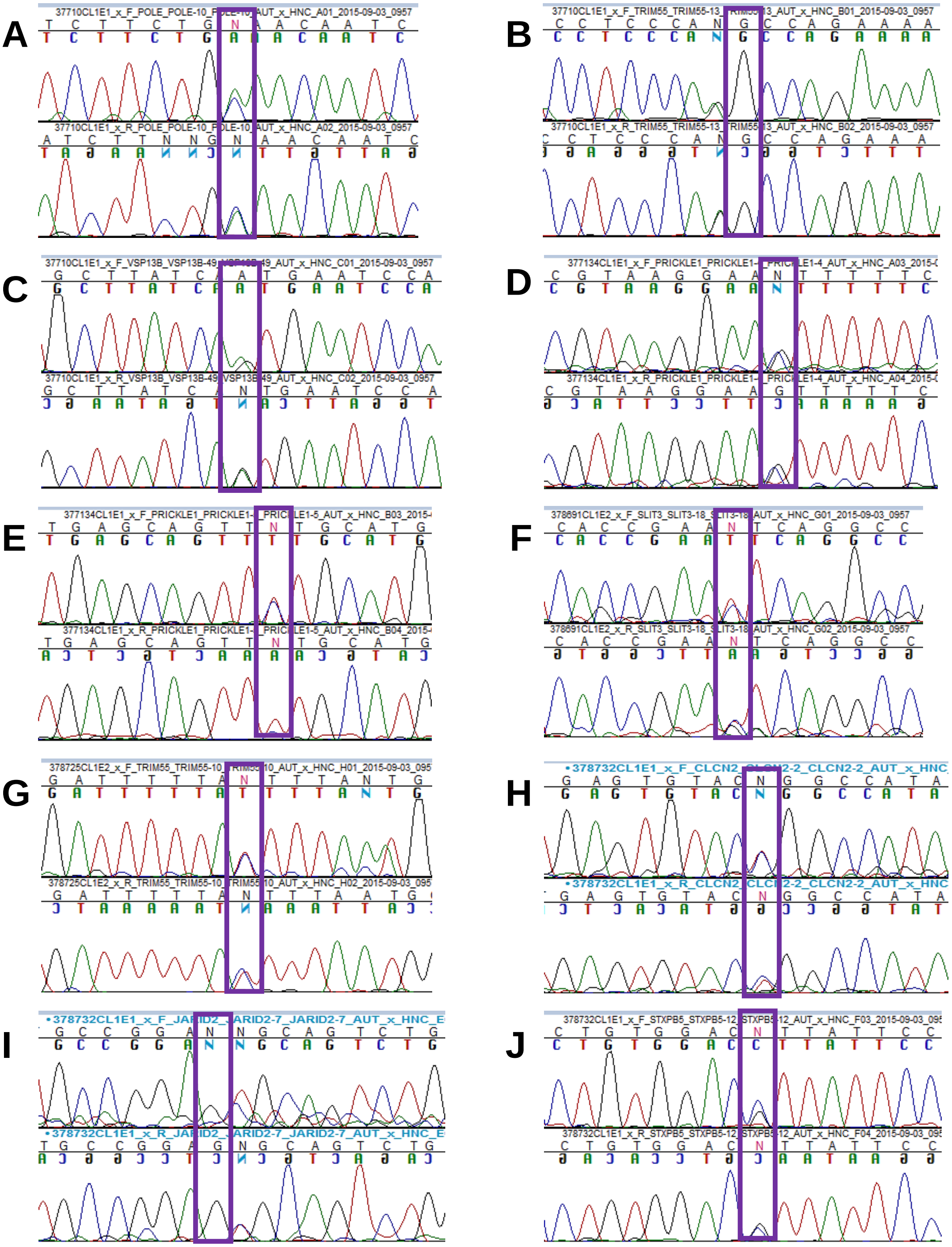

Supplemental Figure 3 | Sanger sequencing validation of the iPSC lines to ensure that the potential ASD alterations are preserved through the reprogramming process. Alterations of interest are enclosed within the purple rectangles and sequencing from both the forward and reverse primers is shown. Cell line 377110 with alterations in POLE at chr12:133252760 (**A**), TRIM55 at chr8:67047224 (**B**), and in VSP13B at chr8:100832259 (**C**). Cell Line 377134 with alterations in PRICKLE1 at chr12:42864125 (**D**) and chr12:42862463 (**E**). Cell line 378691 with an alteration in SLIT3 at chr5:168180047 (**F**). Cell line 378725 with an alteration in TRIM55 at chr8:67067937 (**G**). Cell line 378732 with alteration in CLCN2 at chr3:184076909 (**H**), JARID2 at chr6:15496930 (**I**), and STXBP5 at chr6:147635108 (**J**). All genomic locations are in reference to the Hg19 assembly.

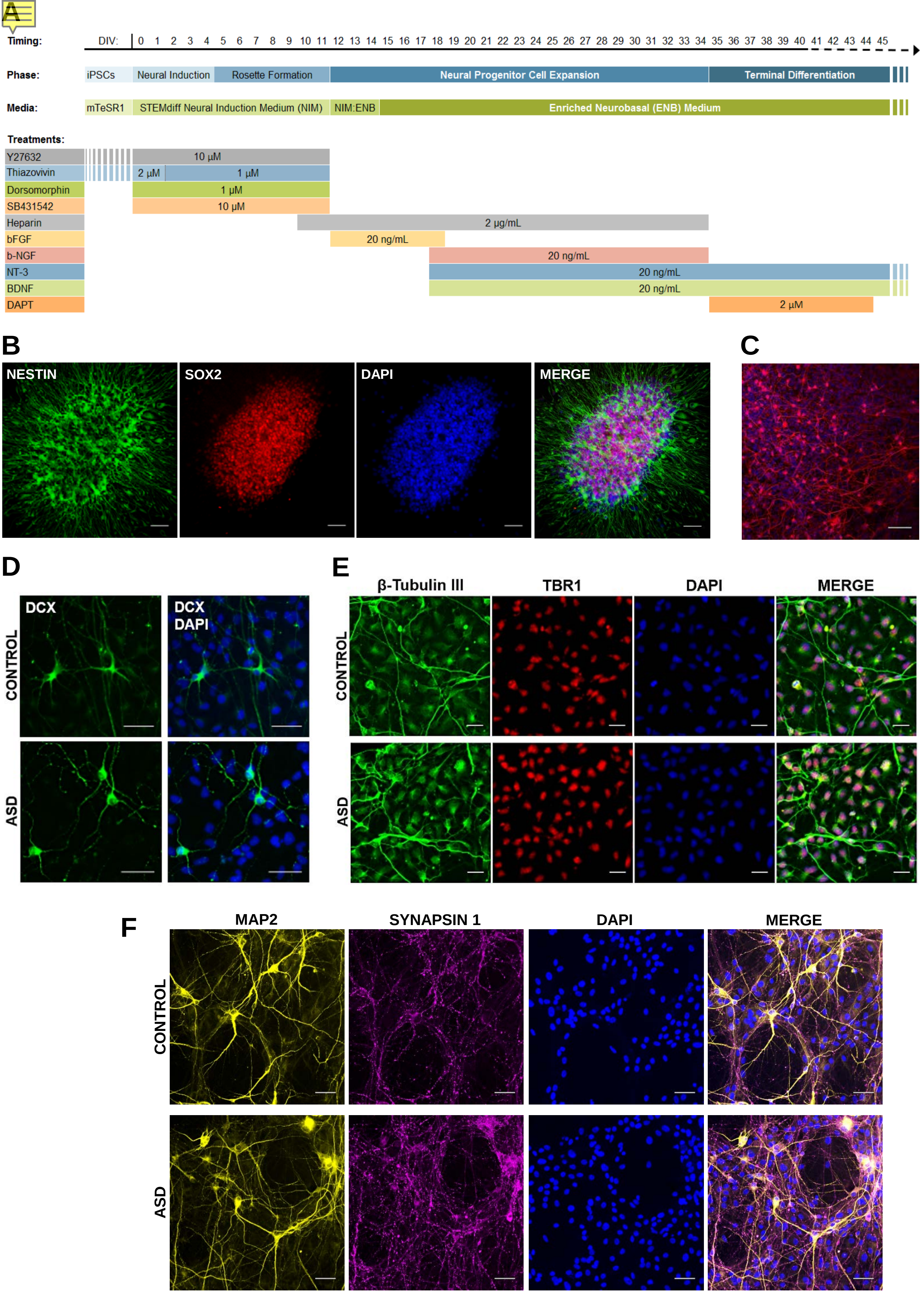

Supplemental Figure 4 | Differentiation of cortical neurons from ASD and control iPSCs.

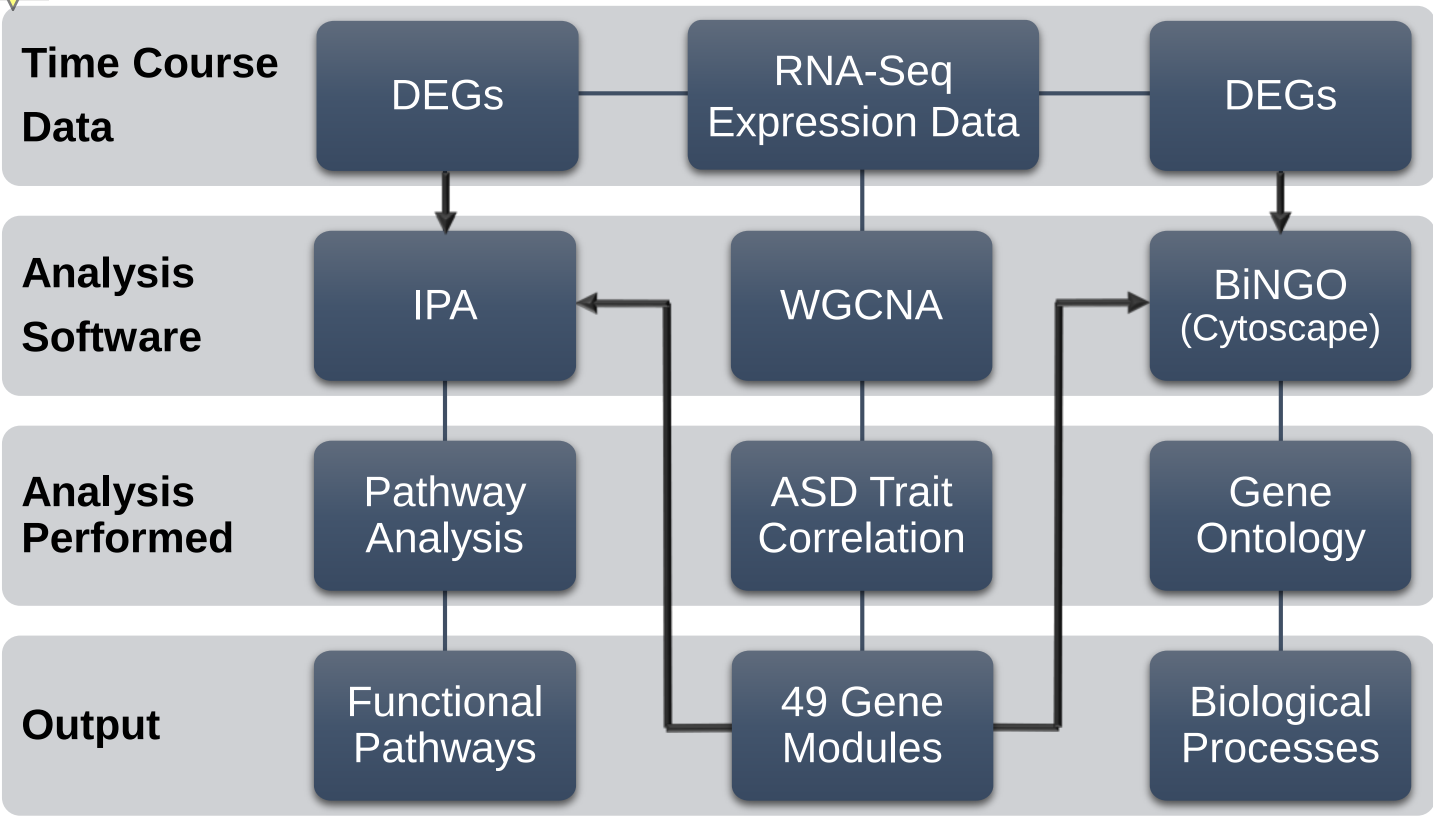

Supplemental Figure 5 | Time course transcriptome data analysis pipelines. ASD and control RNA-Seq data from two different time points (35 and 135 DIV) were fed into single gene (DEGs; identified using EdgeR) and network (WGCNA) analysis pipelines. Note that output generated from WGCNA and differential expression analysis (EdgeR) was fed into IPA and BiNGO analysis software to indicate the pathways and biological processes that are thought to be dysregulated. DIV: Days *in vitro*, IPA: Ingenuity Pathway Analysis (Qiagen), GO: Gene Ontology, WGCNA: Weighted Gene Co-expression Network Analysis, DEGs: Differentially Expressed Genes (FDR<0.05).

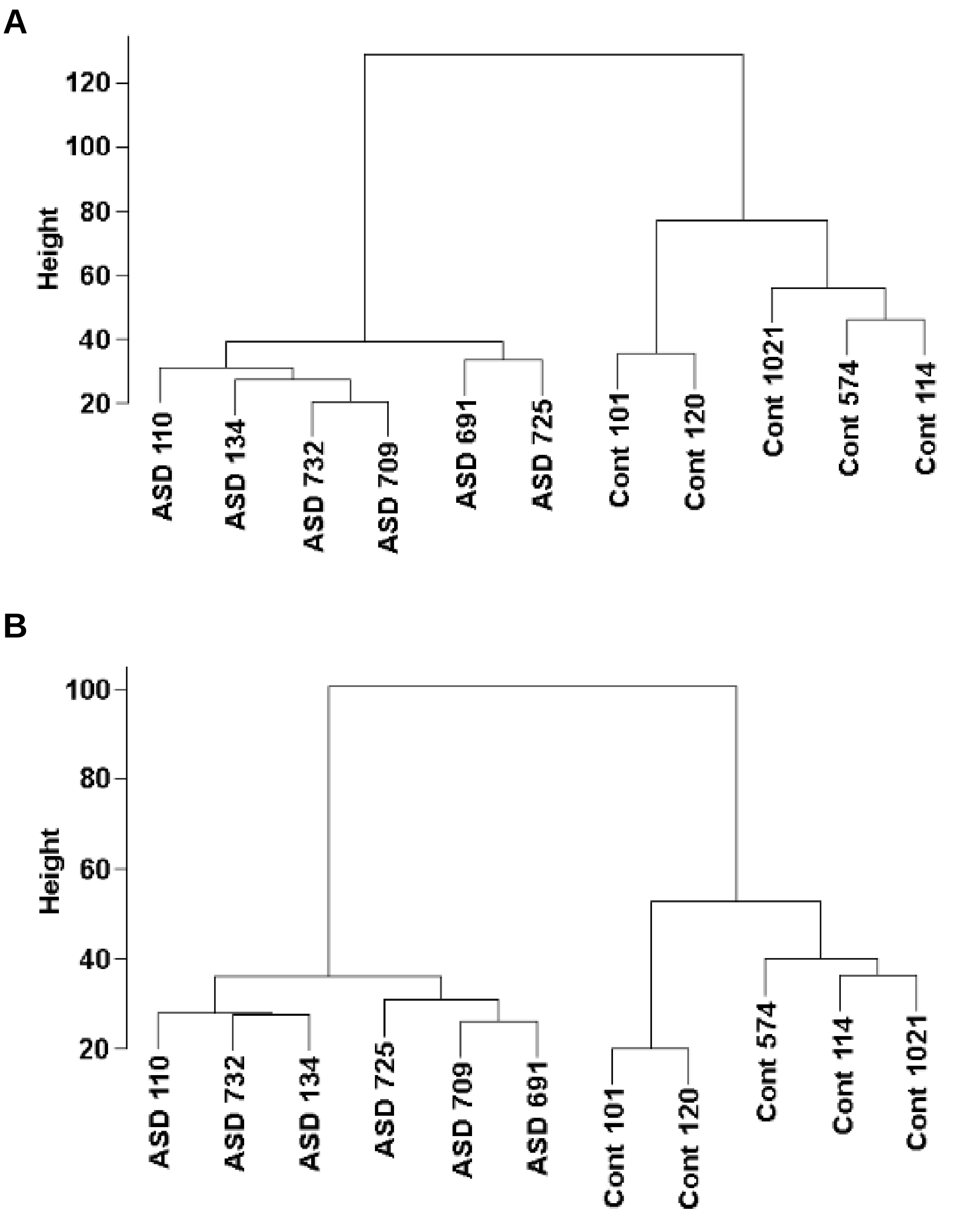

Supplemental Figure 6 | ASD and control iPSC-derived neurons cluster as distinct groups at both the day 35 (A) and day 135 (B) time points based on RNA-seq analysis. Hierarchical clustering of the RNA-seq samples was performed to show the relationship between the samples. The ASD and control samples clearly segregated into distinct groups at both the day 35 (A) and day 135 (B) time points.

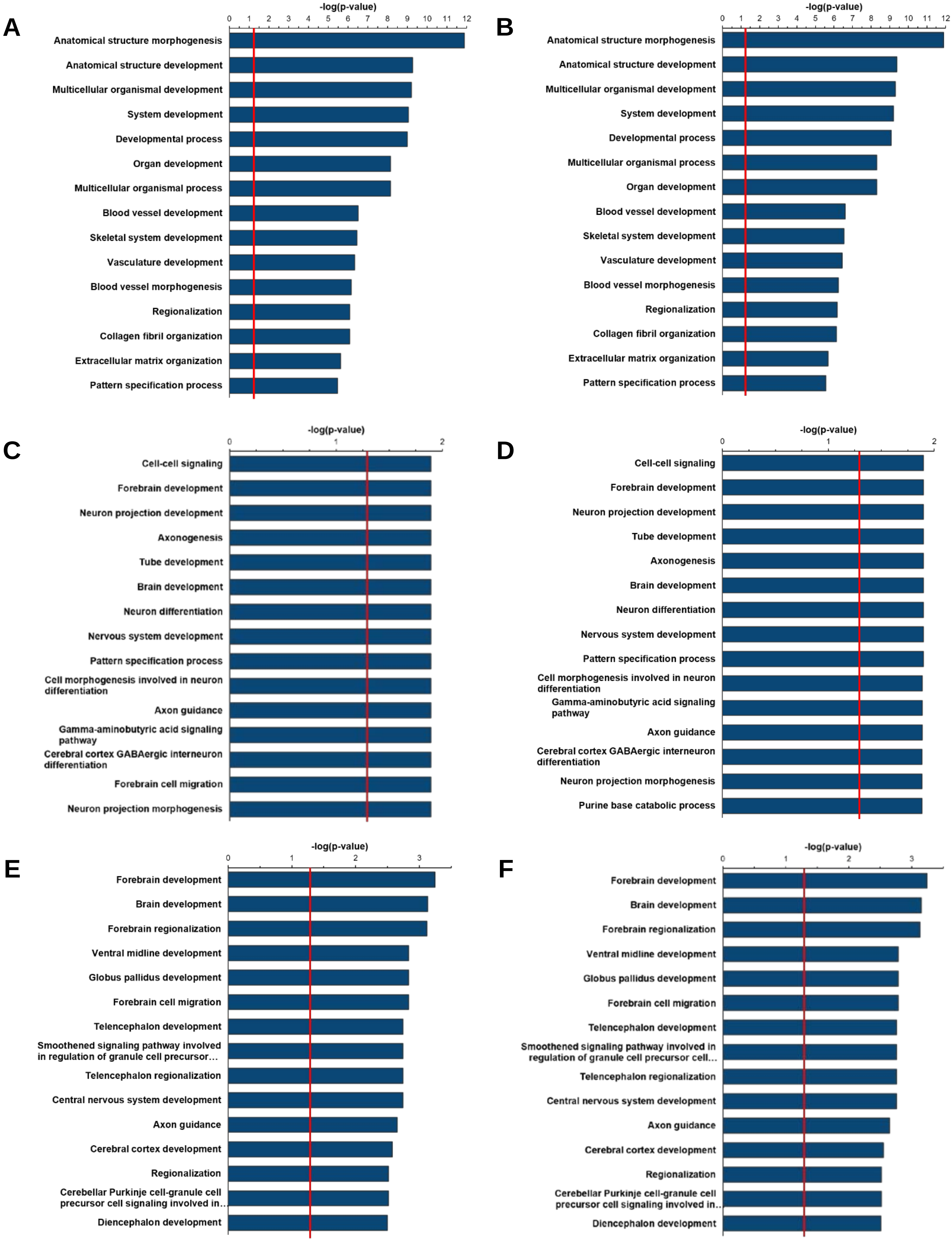

Supplemental Figure 7 | DEG GO biological process enrichments identified using neuronal background sets. Top 15 GO biological processes identified for DEGs using neuronal background sets limited to genes expressed across all iPSC-derived neuronal lines examined in this study (**A**, **C**, **E**) and genes expressed in RNA-Seq BrainSpan samples of human fetal brain (**B**, **D**, **F**). (**A**, **B**) Day 35 DEG biological process enrichments. (**C**, **D**) Day 135 DEG biological process enrichments. (**E**, **F**) Biological processes enriched in DEGs that overlap time points. The red line in the bar plots indicates the cut-off for significance (adjusted P = 0.05).

Supplemental Table 1 | Small molecule and recombinant protein regimen used to differentiate iPSCs into cortical neurons. "Begin" and "omit" column values indicate the day of differentiation that treatments are added/omitted from the culture medium.

Treatment	Concentration	Begin	Omit
Y27632*	10 µM	0	12
Thiazovivin	2 µM	0	2
	1 µM	2	12
Dorsomorphin	1 µM	0	12
SB431542	10 µM	0	12
Heparin	2 µg/mL	10	35
bFGF	20 ng/mL	12	17
β-NGF	20 ng/mL	18	35
NT-3†	20 ng/mL	18	N/A
BDNF†	20 ng/mL	18	N/A
DAPT	2 µM	35	45

*10 µM Y27632 is added to the medium after passaging cells (regardless of day in culture).

†From day 18 and on, NT-3 and BDNF are continuously added to the medium.

Supplemental Table 2 | Primary antibodies used for immunocytochemistry.

Primary Antibodies	Dilutions	Source	Catalog #
Mouse anti-β-Tubulin III (neuronal)	1:100	Sigma	T8578
Guinea pig anti-Doublecortin	1:2000	Millipore	AB2253
Mouse anti-MAP2	1:1000	Gift from Vance Lemmon, LemBix Lab	----
Goat anti-NANOG	1:50	Peirce Thermo Scientific	PA5-18406
Mouse anti-Nestin	1:100	R&D Systems	MAB1259
Mouse anti-Oct 3/4	1:100	STEMCELL Technologies	1550
Mouse anti-SOX2	1:50	R&D Systems	MAB2018
Rabbit anti-Synapsin I	1:500	Millipore	AB1543
Rabbit anti-TBR1	1:200	Abcam	ab31940
Mouse anti-TRA-1-81	1:200	Cell Signaling Technology	4745
